# Personal

**Published:** 1890-04

**Authors:** 


### Personal.

DR. C. B. KIBLER, of Corry, Pa., one of the most celebrated physicians in Western Pennsylvania, paid a visit to Buffalo last month to attend the final examinations of the Buffalo Universiy Medical College, of which he is one of the curators. Dr. Kibler will visit Europe during the coming Summer, expecting to reach Berlin in season to attend the International Medical Congress, after which he will spend some time in the clinics, and return to this country in the late autumn.

---

DR. ROSWELL PARK, of this city, accompanied by Mrs. Park, sailed for Europe, March 19, 1890, for a period of recreation and study. Dr. and Mrs. Charles G. Stockton will follow early in April, and we presume both Drs. Park and Stockton will attend the International Congress in Berlin. They are expected to return in season to resume their professional and college work in the early autumn.

---

Dr. Clinton Cushing, of San Francisco, sailed for Europe on March 4th, to be absent six months. Dr. Cushing will enjoy a much needed rest, renew his acquaintance with his many European friends, attend the International Medical Congress at Berlin, and return in season to participate in the society and college work that begins in the early autumn.

---

Dr. Crego is still at Berlin, and is in the Polyclinic of Prof. Mendal for Nervous Diseases. From here the Doctor goes to Prof. Charcot's Clinic at Paris; from Paris he goes to London, and on his return he will spend a short time in New York at the Roosevelt Nervous Clinic.

